# Differential expression of hypothalamic, metabolic and inflammatory genes in response to short-term calorie restriction in juvenile obese- and lean-prone JCR rats

**DOI:** 10.1038/nutd.2015.28

**Published:** 2015-08-24

**Authors:** A Diane, W D Pierce, R Mangat, F Borthwick, R Nelson, J C Russell, C D Heth, R L Jacobs, D F Vine, S D Proctor

**Affiliations:** 1 Metabolic and Cardiovascular Diseases Laboratory, Molecular and Cell Biology of Lipids Group, Alberta Diabetes and Mazakowski Heart Institutes, Division of Human Nutrition, University of Alberta, Edmonton, Alberta, Canada; 2 Department of Sociology, University of Alberta, Edmonton, Alberta, Canada; 3 Department of Psychology, University of Alberta, Edmonton, Alberta, Canada

## Abstract

**Background:**

Childhood obesity is an important early predictor of adult obesity and associated comorbidities. Common forms of obesity are underpinned by both environmental and genetic factors. However, the rising prevalence of obesity in genetically stable populations strongly suggests that contemporary lifestyle is a premier factor to the disease. In pediatric population, the current treatment/prevention options for obesity are lifestyle interventions such as caloric restriction (CR) and increase physical activity. In obese individuals, CR improves many metabolic parameters in peripheral tissues. Little is known about the effect of CR on the hypothalamus. This study aimed to assess the effect of CR on hypothalamic metabolic gene expression of young obese- and lean-prone animals.

**Methods:**

Male juvenile JCR:LA-cp obese-prone rats were freely fed (Obese-FF) or pair fed (Obese-FR) to lean-prone, free-feeding animals (Lean-FF). A group of lean-prone rats (Lean-FR) were matched for relative average degree of CR to Obese-FR rats.

**Results:**

In free-feeding conditions, obese-prone rats consumed more energy than lean-prone rats (*P*<0.001) and showed greater increases in body weight, fat mass, plasma glucose, insulin and lipids (*P*<0.01). These metabolic differences were associated with alterations of feeding-related neuropeptides expression in the hypothalamus, as well as pro-inflammatory cytokines and oxidative stress markers. When submitted to the same degree of CR, the two genotypes responded differently; hypothalamic inflammatory and oxidative stress gene expression was improved in Obese-FR, while it was worsened in Lean-FR rats.

**Conclusions:**

We demonstrate in JCR rats that the metabolic and inflammatory response of the brain to CR is genotype dependent.

## Introduction

Over the last few decades, the incidence of pediatric obesity has increased exponentially in developed countries and developing countries in economic transition.^1^ Childhood obesity is an important early predictor of adult obesity^2^ and its associated chronic co-morbidity risks, including cardiovascular disease^3^ and type 2 diabetes.^4, 5^ Although the etiology of obesity is not well understood, evidence suggests that common forms of obesity are underpinned by both environmental and genetic factors.^6, 7^ However, the rising prevalence of obesity in genetically stable populations also suggests that contemporary lifestyle such as calorie-abundant foods and reduced physical activity is a main contributing factor to the disease.^8^ This observation suggests the need to tackle the environmental factors leading to obesity. Within the pediatric population, lifestyle modifications such as caloric restriction (CR) and increased physical activity are the first-line approach for the prevention or treatment of obesity.^9^

Body weight is affected by biological, environmental and behavioral factors, all of which are influenced by genetic background.^10, 11^ For the obese-prone phenotype, CR without malnutrition improves many metabolic and inflammatory parameters in peripheral tissues.^12, 13^ However, the effects of CR on the hypothalamus, the ‘headquarters' in the regulation of many fundamental physiological activities, including metabolic and energy homeostasis^14^ are not well documented.

In the hypothalamus (and mostly within the arcuate nucleus (ARC)), the mechanisms of energy-balance regulation involve the interaction of multiple neuropeptides including orexigenic peptides such as neuropeptide Y (NPY), agouti-related protein and hypocretin/orexin, as well as anorectic peptides such as pro-opiomelanocortin, cocaine–amphetamine-regulated transcript and corticotropin-releasing hormone. These orexigenic and anorxigenic peptides are altered in both obesity induced by leptin signaling deficiency^15^ and CR.^16^

Chronic overfeeding associated with accumulation of adipose tissue is known to trigger an atypical form of inflammation and oxidative stress, which lead to metabolic dysfunctions at cellular, organ and systemic levels.^17, 18^ In addition, intracellular inflammation and related oxidative stress generated by overfeeding are linked to metabolic syndrome via the hypothalamus^19, 20^—impairing hypothalamic neurons involved in energy balance and feeding, and disrupting central adiposity sensitivity to leptin and insulin.^20^ On the other hand, chronic severe food restriction and depletion of fat stores also generate brain inflammation and oxidative stress. Owing to the role of central inflammation in metabolic syndrome initiation,^21^ it is possible that animals with chronic severe food restriction-induced brain inflammation can be well predisposed to metabolic syndrome under conditions of plenty, suggesting a vicious cycle between food availability, brain inflammation and metabolic syndrome. Therefore, the differential response of the brain to calorie availability may relate to genetic differences, duration of CR and/or the degree of caloric deprivation employed.^22^ The objective of the present study was to determine the levels of hypothalamic feeding-related neuropeptides, inflammation and oxidative stress gene expression of obese-prone and lean-prone juvenile JCR rats submitted to a similar degree of CR.

We hypothesized that young obese-prone animals under free-feeding conditions would display higher expression of hypothalamic appetite-regulatory, inflammatory and oxidative stress genes than their lean-prone counterparts. Furthermore, we hypothesized that obese- and lean-prone rats matched on CR would show differential expression of these genes in the hypothalamus.

## Materials and methods

### Animal model

Twenty-four male JCR:LA-cp rats with an obese-prone phenotype (corpulent trait carriers: cp/cp) and 12 control rats with a lean-prone phenotype (non-expressing and non-corpulent trait carriers: +/?) 35–40 days of age (juveniles) were obtained from our established breeding colony at the University of Alberta.^23^ The JCR-LA-cp rats express a corpulent trait (cp) due to an autosomal recessive, nonsense Tyr763Stop mutation in the *Ob-R* gene, resulting in a total absence of functional leptin receptors.^24^ Thus, rats that are homozygous for the cp trait (cp/cp) are obese prone and display metabolic syndrome characteristics observed in human obesity, including hyperphagia, hyperinsulinemia and hyperlipidemia, which can be observed at 4 weeks of age;^25^ those that are heterozygous for the cp trait (+/cp) or wild type (+/+) are lean prone. As the wild type (+/+) and the heterozygous (cp/+) are both lean prone and are physically undistinguishable, they are represented as (+/?). The ‘+/?' means a 2:1 mix of cp/+ (heterozygote) and +/+ (homozygous wild type). The study was conducted in two separate batches (12 rats per batch) with 6 obese-prone (cp/cp) and 6 lean-prone (+/?) animals in each batch. The rats were housed individually in clear polycarbonate cages (47 cm × 27 cm × 20 cm) with sterile wood chip bedding in a temperature- (22±2 °C) and humidity-controlled environment and maintained on a 12 h light/dark cycle (lights off 1000–2200 h). Throughout the experiment, animals had free access to water and were fed as outlined in the experimental procedure. The care and use of animals was in accordance with the Guidelines of the Canadian Council of Animal Care and subject to prior review and approval by the University of Alberta Animal Care and Use Committee: Livestock.

### Experimental procedure

One day after arrival, rats were measured for lean and fat body mass using nuclear magnetic resonance (Minispec LF90 Body Composition Analyzer; Bruker, East Milton, ON, Canada). Obese-prone rats were matched for body weights and randomly assigned to free feeding (Obese-FF) or food-restricted (Obese-FR) groups (six rats per group). Lean-prone animals were randomly assigned to free-feeding (Lean-FF) or food-restricted (Lean-FR) groups (six rats per group). In obese-prone rats, CR consisted of pair feeding the obese-prone rats the daily mean amount of food consumed by the Lean-FF group from the previous day. The daily food intake of Lean-FR rats was a percentage of the food consumption of Lean-FF animals; we calculated this percentage from the difference in daily food intake of Obese-FF and Obese-FR groups (Lean-FR intake=Lean-FF intake*(Obese-FF intake−Obese-FR intake)/Obese-FF intake*100). Thus, Obese-FR and Lean-FR groups were matched on relative average amount of CR. Animals were fed standard laboratory chow (LabDiet 5010 Rodent Diet, PMI Nutrition International Inc., Brentwood, MO, USA). An electronic scale (Sartoris, Model TE4101, Sartorius AG, Goettingen, Germany) was used to measure daily food and body weight to the nearest gram.

### Post-mortem analyses

After 10 days on the feeding schedule, rats were removed from their home cages, fat and lean body mass were measured again as at baseline and then animals were anesthetized with isoflorane in oxygen. Blood was taken by cardiac puncture, transferred to 10-ml polyethylene tubes containing EDTA and stored on ice until centrifugation (3900 *g*, 10 min). Plasma samples were stored at −80 °C until analysis. The rats were immediately perfused intracardially with ice-cold isotonic saline: the brain was removed and immediately frozen before sectioning, as described below. Lean body mass and white adipose tissue were expressed as a percentage of total body weight.

### Brain microdissection

Cryostat (Model tissue Tek II, Miles, Minneapolis, MN, USA) at an approximate operating temperature of −20 °C was used for coronary sections from frozen rat brains. We focused mostly on the ARC, owing to its close proximity to the peripheral blood stream and its strong response to peripheral hormonal and nutritional signals. Punches containing this targeted area of the brain were taken based on The Rat Brain Stereotaxic Coordinates^26^. Punches were stored at −80 °C until further RNA extraction processing.

### Hypothalamic gene expression

An array of target genes (*n*=44) was measured by performing real-time quantitative PCR using a high-throughput method (Biomark Fluidigm System, San Francisco, CA, USA) with primers and corresponding universal probe library (Roche Applied Science, Laval, QC, Canada) probes designed with the Roche UPL design centre software as previously described^27^ (Supplementary Table S1). Total RNA was isolated from frozen ARC tissues using TRIzol (Invitrogen, Burlington, ON, Canada) as described in the manufacturer's protocol, and further cleaned up using RNEasy MinElute Cleanup kit columns (Qiagen, Toronto, ON, Canada). RNA integrity was checked with Bioanalyzer chip analysis (Agilent, Mississauga, ON, Canada). RNA was reverse transcribed into cDNA using the High-Capacity cDNA Reverse Transcription kit (Applied Biosystems, Mississauga, ON, Canada). Forty-eight gene assays (44 target genes+2 housekeeping genes in duplicate) and cDNA samples were loaded into separate wells on a 48.48 format gene expression chip (Biomark Fluidigm System). Target gene expression was normalized to the housekeeping gene cyclophilin and fold changes relative to control were calculated using the comparative *C*_T_ (2^-ΔΔCt^) method. All assays were performed in triplicate. With the 44 target genes assessed, we are only reporting the gene expression values mainly involved in appetite regulation, inflammation and oxidative stress; the remaining gene expression values are available in the Supplementary Table S2.

### Plasma biochemical analysis

Total plasma cholesterol and triglyceride concentrations were measured using colorimetric chemical enzymatic kits (Wako Chemicals USA, Inc., Richmond, VA, USA). Plasma glucose was determined using a glucose oxidase method (Diagnostic Chemical Ltd, Charlottown, PEI, Canada, catalog number 220-32). Insulin and leptin levels were assessed by enzyme-linked immunosorbent assay kit for rodents (insulin kit, Mercodia AB, Uppsala, Sweden, and leptin kit, LINCO Research, St. Charles, MO, USA).

### Statistical analyses

Results are presented as means±s.e.m. and were plotted using GraphPad (Prism v5.0a, La Jolla, CA, USA). We used *t*-tests (paired or independent samples) and two-way analysis of variance to test genotype (obese-prone vs lean-prone) by feeding condition (free access vs CR) effects and one-way analysis of variance for comparison of the four groups with *post-hoc* Tukey's test for pairwise comparisons, and *P*<0.05 were considered statistically significant. For multiple comparisons of means tests, the least significance *α*-level for the several contrasts is reported in the text.

## Results

### Energy intake and body weight

**Figure 1:**
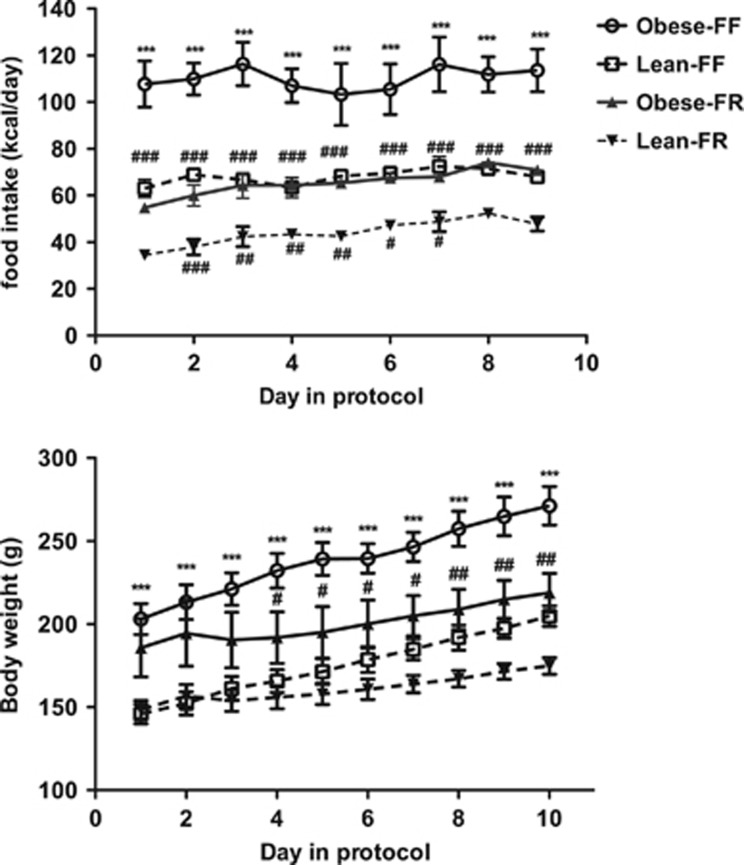
Energy intake (upper panel), body weight (lower panel) in the JCR:LA-cp obese-prone and lean-prone rats following caloric restriction (*n*=6 rats per group). Data are shown as mean±s.e.m. ***P<0.001 Lean-FF compared with Obese-FF; #*P*<0.05, ##*P*<0.01, ###*P*<0.001 Obese-FF compared with Obese-FR or Lean-FF vs Lean-FR. FF, free feeding; FR, food restricted.

Daily caloric consumption over the period is shown in Figure 1 (upper panel), together with body weight, Figure 1 (lower panel). As expected, energy intake was different between groups over the 10-day period. There was a time (day) by feeding condition interaction on caloric consumption (*P*=0.03), but no time by genotype interaction. Not surprisingly, juvenile free-fed rats consumed more calories than CR animals over the 10-day period (*P*<0.01). Obese-prone rats also consumed more calories than lean-prone animals, yielding a genotype effect (*P*<0.01; Figure 1, upper panel). Notably, Lean-FR and Obese-FR juvenile rats did not significantly differ in percentage daily caloric intake compared with their free-feeding controls. The mean daily caloric intake of Lean-FR rats as a percentage of the Lean-FF was 64.69±2.16%, and that of Obese-FR rats was 60.21± 2.03% of Obese-FF intake. Thus, the two CR groups showed no statistical difference in relative caloric intake.

There was a significant time by feeding interaction (*P*<0.001) in body weight (Figure 1, lower panel), with free-fed juvenile rats having greater body weight than CR rats over 10 days. There was also a significant genotype effect; obese-prone rats weighted more than lean-prone animals (obese prone=219.66±6.93 g and lean prone=168.28±6.63 g; *P*<0.001) and a significant feeding condition effect. Free-fed juvenile rats had increased body weight compared with CR rats (207.11±6.93 vs 180.82±6.63 g; *P*=0.014). After 10 days of feeding, Obese-FF rats weighted more than Obese-FR, Lean-FF or Lean-FR rats (*P*<0.001). Obese-FR and Lean-FF rats did not significantly differ in their absolute body weight, indicating that pair feeding of obese-prone rats to lean-prone animals normalized the body weight of juvenile animals. However, Lean-FR and Lean-FF rats did not significantly differ in their absolute body weight after 10 days of pair feeding despite that Lean-FR ate 64% of the intake of the Lean-FF rats. A trend analysis showed a difference in linear trend (*P*<0.01), indicating that juvenile Lean-FF increased body weight more over days than Lean-FR rats of the same age (Figure 1; lower panel).

### Body composition and plasma biochemical parameters

**Figure 2:**
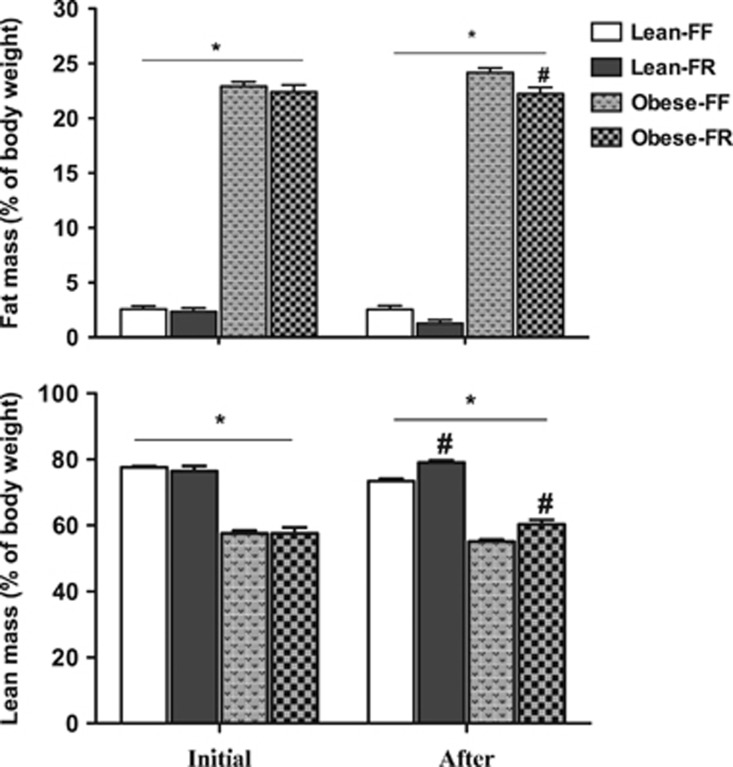
Body composition: percentage of fat mass and lean body mass in the JCR:LA-cp obese-prone and lean-prone rats before (initial) and after 10 days of CR (*n*=6 rats per group). Data are shown as mean±s.e.m. *P<0.05 Lean-FF compared with Obese-FF; #*P*<0.05 Obese-FF compared with Obese-FR or Lean-FF vs Lean-FR. FF, free feeding; FR, food restricted.

Figure 2 depicts the body composition of the animals before and after the pair-feeding intervention. Initially, as expected, juvenile obese-prone rats with no leptin receptor functional had a higher percentage of fat than lean-prone animals of similar age (*P*<0.001), while the opposite was found for the percentage of the lean body mass. CR induced by pair feeding significantly improved body composition. Caloric-restricted animals had significantly less fat mass than free-fed rats (*P*=0.02) and greater lean body mass than free-fed animals (*P*< 0.05).

Table 1 shows the biochemical parameters. Lean-FF rats had significantly lower concentrations of plasma triglyceride (*P*<0.001), total cholesterol (*P*<0.001), glucose (*P*<0.001) and insulin (*P*<0.001) than the Obese-FF rats. Similar degree of CR significantly reduced triglyceride in both genotypes, while glucose and insulin levels were reduced significantly only in the obese-prone rats (*P*<0.05).

### ARC neuropeptide and inflammatory mRNA expression

**Figure 3:**
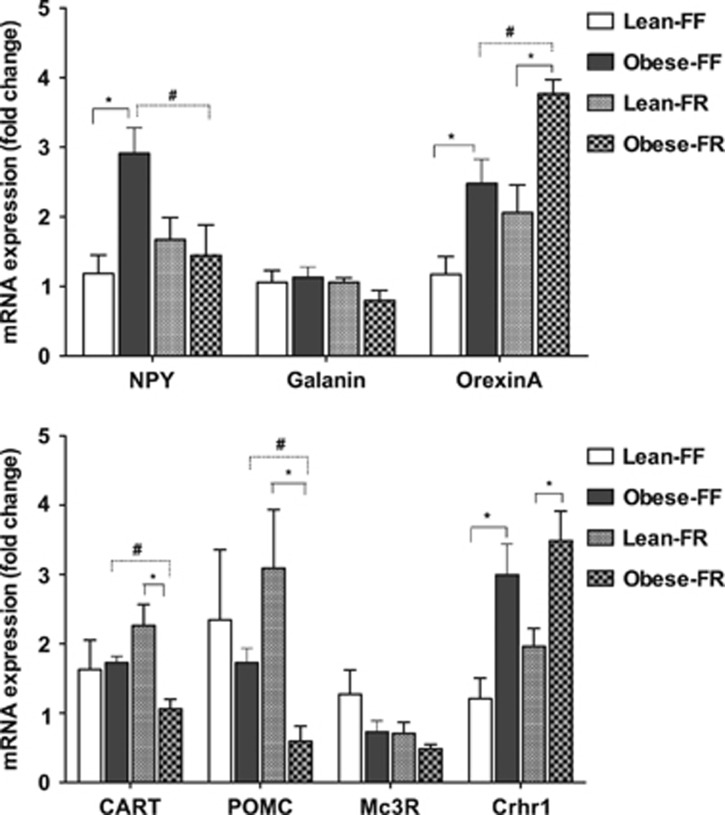
Hypothalamic ARC mRNA expression of orexigenic (upper panel) and anorexigenic (lower panel) neuropeptides in the JCR:LA-cp obese-prone and lean-prone rats following caloric restriction (*n*=6 rats per group). Data are shown as mean±s.e.m. *P<0.05 Lean-FF compared with Obese-FF; #*P*<0.05 Obese-FF compared with Obese-FR or Lean-FF vs Lean-FR. FF, free feeding; FR, food restricted. NPY, neuropeptide Y; POMC, pro-opiomelanocortin; CART, cocaine and amphetamine-regulated transcript; Mc3R, melanocortin receptor 3; Crhr1, corticotropin-releasing hormone receptor 1. Target gene expression was normalized to a reference gene (cyclophilin).

There was no significant difference in the hypothalamic expression levels of the orexigenic neuropeptide galanin between the four experimental groups (Figure 3, upper panel). In the free-feeding condition, NPY and Orexin A mRNA levels were significantly (+142% and +107%, respectively) higher in the juvenile obese-prone rats, compared with lean-prone animals of similar age (*P*<0.05). This suggests that the leptin pathway may downregulate these neuropeptides in juvenile free-feeding animals. In lean-prone juvenile rats, CR had no significant effect on the expression of NPY and Orexin A. In obese-prone juvenile rats, CR significantly decreased NPY mRNA (*P*<0.05) and increased Orexin A mRNA (*P*<0.05) levels (Figure 3, upper panel). Under the free-feeding condition, there were no significant differences in cocaine–amphetamine-regulated transcript and pro-opiomelanocortin expressions between the obese-prone and lean-prone groups (Figure 3, lower panel). However, under a similar degree of relative CR, lean-prone juvenile rats displayed higher levels of cocaine–amphetamine-regulated transcript and pro-opiomelanocortin mRNA than obese-prone animals of similar age, suggesting that the presence of the leptin pathway upregulates these anorexigenic neuropeptides during CR of juvenile rats. In both feeding conditions (free feeding and food restriction), there was a genotype effect on CRHR1 mRNA expression; obese-prone rats showed significantly higher CRHR1 expression than lean-prone animals (*P*<0.05; Figure 3, lower panel).

**Figure 4:**
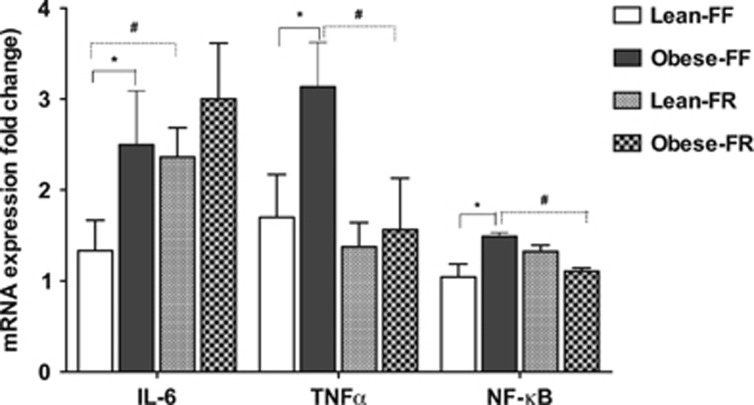
Hypothalamic ARC mRNA expression of inflammatory genes in the JCR:LA-cp obese-prone and lean-prone rats following CR (*n*=6 rats per group). Data are shown as mean±s.e.m. *P<0.05 Lean-FF compared with Obese-FF; #*P*<0.05 Obese-FF compared with Obese-FR or Lean-FF vs Lean-FR. FF, free feeding; FR, food restricted; IL-6, interleukin-6; TNFα, tumor necrosis factor-α NF-κB, nuclear factor-κB. Target gene expression was normalized to a reference gene (cyclophilin).

**Figure 5:**
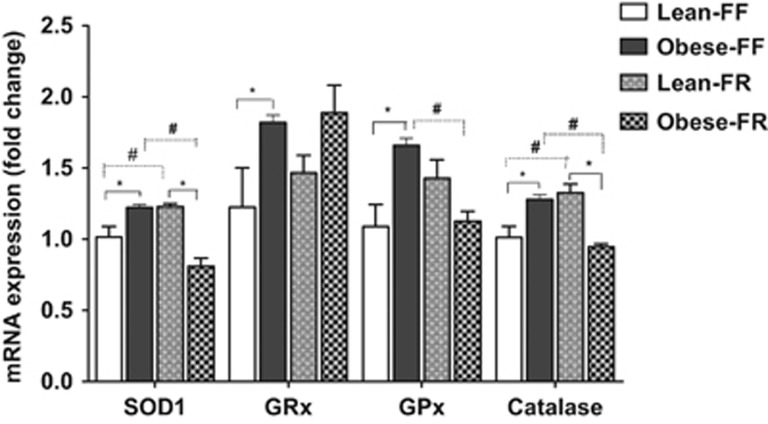
Hypothalamic ARC mRNA expression of oxidative stress genes in the JCR:LA-cp obese-prone and lean-prone rats following CR (*n*=6 rats per group). Data are shown as mean±s.e.m. *P<0.05 Lean-FF compared with Obese-FF; #*P*<0.05 Obese-FF compared with Obese-FR or Lean-FF vs Lean-FR. FF, free feeding; FR, food restricted; SOD1, superoxide dismutase 1; GRx, glutathione reductase; GPx; glutathione peroxidase. Target gene expression was normalized to a reference gene (cyclophilin).

Figure 4 shows the inflammatory markers by genotype and feeding conditions. Under free-feeding conditions, obese-prone rats displayed significantly higher interleukin-6, tumor necrosis factor-α and nuclear factor-κB mRNA levels in the ARC than lean-prone animals, indicating downregulation of expression of these cytokines by the leptin pathway in juvenile free-feeding rats (*P*<0.05). Pair feeding significantly lowered these pro-inflammatory cytokine expression levels in juvenile obese-prone rats. As shown in Figure 4, average expression levels for the Obese-FR group were significantly lower than Obese-FF rats (*P*<0.05). In lean-prone juvenile rats, a similar degree of relative CR had no significant effect on tumor necrosis factor-α and nuclear factor-κB mRNA expression. Lean-FR rats had significantly increased expression of interleukin-6 compared with the Lean-FF rats (*P*<0.05, Figure 4). Under conditions of free access to food, the ARC expression of oxidative stress markers, as shown in Figure 5, was significantly higher in the juvenile obese-prone rats as compared with the lean-prone animals of similar age (*P*<0.05). Interestingly, pair feeding of obese-prone to lean-prone rats significantly reduced the hypothalamic expression levels of superoxide dismutase-1, glutathione reductase, glutathione peroxidase and catalase mRNA in Obese-FR rats as compared with the Obese-FF group (*P*<0.05). In lean-prone rats, however, with the same degree of relative CR, the expression of superoxide dismutase-1 and catalase mRNA in the ARC was significantly increased compared with the Lean-FF rats (*P*<0.05; Figure 5).

## Discussion

Our results show for the first time that feeding-related neuropeptides, inflammation and related oxidative stress are altered in the hypothalamus of juvenile obese-prone JCR rats before the overt onset of obesity. In pre-pubertal JCR rats (35–40 days of age), obese-prone rats, with defective leptin signaling, showed higher hypothalamic NPY and Orexin A mRNA expression than lean-prone animals of similar age (with intact leptin receptor), but no difference in pro-opiomelanocortin and cocaine–amphetamine-regulated transcript expression under the free-feeding condition. Leptin is known to regulate arcuate hypothalamic neurons directly by binding to the ObR receptor long isoform, activating Janus kinases (JAK) and (3) signal transducer and activator of transcription (STAT) signaling and suppressing the activation of NPY neurons.^28^ Thus, NPY overexpression observed in the juvenile obese-prone rats could be a result of an absence of functional leptin receptors.

The upregulation of orexigenic neuropeptides (NPY and Orexin A) in leptin receptor-deficient rodents is often accompanied by physiological and metabolic changes.^29^ With free access to food, the energy intake of juvenile obese-prone rats (with no functional leptin receptor) was consistently higher than that of lean-prone animals of similar age. This greater energy consumption of obese-prone juvenile animals, a consequence of non-functional leptin receptor, was associated with greater gains in body and fat mass weight, and increased plasma glucose, insulin and lipid concentrations. These findings are in agreement with previous studies.^23^ The lower fat depot found in the juvenile lean-prone rats compared with the obese-prone JCR counterparts is a result of leptin action on energy balance. Leptin is also known to induce expression of uncoupling proteins (UCP-1/-2/-3) in the mitochondria via stimulation of β3-adrenaergic receptors, thereby leading to increased thermogenesis^30^ and hence reduced fat deposition.

The link between overfeeding, hypothalamic inflammation and the control of body weight is fundamental to how insulin resistance develops when tissues are exposed to a supply of nutrients that exceeds their energy requirements.^20, 31^ In the present study, under free access to food, the increased hypothalamic inflammation observed in the juvenile obese-prone rats, with greater food consumption, could be a consequence of excessive nutrients presented to the hypothalamus, as previously reported.^21^ Moreover, De Souza *et al.*^32^ have found that high-fat feeding (lipidemic diet) induces a local pro-inflammatory status in the hypothalamus, which results in impaired anorexigenic insulin signaling. In the current study, our results show that under regular chow regime, the overeating of the obese-prone JCR juvenile rats promptly activates inflammatory and related oxidative stress pathways in the hypothalamus. Compared with lean-prone rats of similar age, obese-prone animals showed greater interleukin-6, tumor necrosis factor-α and nuclear factor-κB mRNA expression in the ARC, as well as increased expression of markers of oxidative stress (glutathione reductase, glutathione peroxidase and catalase). This finding is in line with a previous report that revealed increased microglial activation or neuroinflammation associated with obesity in JCR rats using positron emission tomography imaging.^33^ However, the underlying mechanism(s) of overfeeding-induced hypothalamic inflammation remain elusive.

Recent research, using genetic and molecular approaches, suggests that overnutrition-induced hypothalamic metabolic inflammation involves at least three levels of responses consisting of (i) membrane receptor-independent intracellular stresses^34, 35^, (ii) the Toll-like receptor pathway^36^ and (iii) the cytokine/chemokine receptor pathways.^37^ Compared with classical (for example, pathogen-induced) inflammation, inflammation triggered by nutrient excess is termed ‘metabolic inflammation.'^38^ More importantly, unlike inflammation in peripheral tissues, hypothalamic inflammation induced by nutrient excess impairs neuronal sensitivity to leptin and insulin stimulation, which consequently leads to obesity development by altering feeding-related neuropeptides.^21^ In addition, this high inflammatory response induced by overnutrition in juvenile obese-prone JCR rats (before an overt obesity phenotype) associated with orexigenic neuropeptides upregulation could also participate in overt metabolic syndrome development during adulthood.

In addition to the negative outcome associated with obese-prone genotype under free-fed conditions, our study is also the first to illustrate that under a similar degree of CR, obese-prone and lean-prone JCR rats respond differently. Submitting juvenile obese-prone and lean-prone JCR rats to a similar degree of CR reduced plasma triglyceride concentrations and total fat mass, compared with freely fed controls. The lower plasma lipids and fat mass in the CR rats are associated with higher Orexin A expression rather than NPY expression in these animals. CR is known to increase NPY expression that triggers food intake.^29^ However, owing to the experimental procedure, food availability is intermittent; hence, the catabolic pathway is also activated to produce energy, leading to the reduction of white adipose tissue in the CR animals.

This physiological adaptation explains the Orexin A upregulation in the food-restricted animals, as this neuropeptide is more likely to be involved in the control of energy metabolism rather than in caloric intake^39^ stimulating lipolysis and increasing β-oxidation.^40^ We also found that hypothalamic pro-inflammatory cytokines and oxidative stress markers were significantly reduced in the obese-prone juvenile rats after 10 days of CR, whereas a similar degree of CR significantly increased these parameters in the lean-prone juvenile rats for the same period. The high level of hypothalamic pro-inflammatory cytokines observed in lean-prone rats following the CR relative to the free-feeding animals is in agreement with recent report showing that food restriction significantly increases hypothalamic expression of tumor necrosis factor-α in Wistar rats,^41^ suggesting that in metabolically normal conditions, CR induces inflammatory stress, which might contribute to the onset of hypothalamic inflammation. Indeed, owing to the lower percentage of fat mass in the lean-prone rats, 36% of CR induced with the experimental protocol may be functionally defined as a severe CR that most likely induced physiological stress, which may contribute to the onset of hypothalamic inflammation as previously reported.^42, 43^ Thus, taken on the account of the importance of central inflammation in the regulation of energy homeostasis, it cannot be ruled out that the increased hypothalamic inflammation observed in the Lean-FR rats may likely lead to the development of metabolic syndrome when food becomes freely available.

In conclusion, our findings report for the first time that under free-feeding conditions, the obese-prone genotype based on leptin receptor deficiency was detrimental for juvenile animals, inducing overexpression of hypothalamic orexigenic feeding-related neuropeptides and inflammatory genes associated with hyperphagia and body weight and fat mass gains. However, under a similar degree of CR the two genotypes (lean-prone vs obese-prone) respond differently; CR improves hypothalamic inflammation and oxidative stress in juvenile obese-prone rats, but aggravates these markers in lean-prone animals of similar age. Clinical implications of these findings lie in supporting the rationale for incorporating genotyping into obesity management based on CR.

## Figures and Tables

**Table 1:** Biochemical parameters in obese-prone and lean-prone JCR:LA-cp rats following CR

	*Lean-prone rats*		*Obese-prone rats*	
	*Lean-FF*	*Lean-FR*	*Obese-FF*	*Obese-FR*
Triglycerides (mg dl^−1^)	77.1±3.1^a^	29.6±2.4^b^	244.3±21.7^c^	134.8±27.8^d^
Cholesterol (mg dl^−1^)	76.3±1.5^a^	58.2±1.3^b^	126.6±13.2^c^	96.8±6.7^c^
Glucose (mg dl^−1^)	140.1±5.6^a^	139.3 ±10.7^a^	206.1±6.7^b^	149.4±12.0^c^
Insulin (ng ml^−1^)	1.7±0.9^a^	ND	16.7±8.7^b^	4.7±1.4^c^

Abbreviations: ANOVA, analysis of variance; CR, caloric restriction; FF, free fed; FR, food restricted; Lean-FF, lean free feeding; Lean-FR, lean food restricted; ND, non-detected; Obese-FF, obese free feeding; Obese-FR, obese food restricted.

Values are mean±s.e.m. The superscripts a, b, c and d are used to denote statistical differences among groups. Within the same row, means with different superscripts (corresponding to the results from follow-up tests using one-way ANOVAs with *post-hoc* comparisons) are significantly different (*P*<0.05).
