# Supplementary material for: Differential expression of hypothalamic, metabolic and inflammatory genes in response to short-term calorie restriction in juvenile obese- and lean-prone JCR rats
Source: Nutr Diabetes. 2015 Aug 24;5(8):e178–. doi: 10.1038/nutd.2015.28 (PMC4558559; doi:10.1038/nutd.2015.28)
Supplement: Supplementary Table 1 [file nutd201528x1.pdf]

Table 1 Supplement: List of target and housekeeping genes.

	Forward primer	Reverse primer	Roche UPL Probe
Cyclophilin	TGCTGGACCAAACACAAATG	CTTCCCAAAGACCACATGCT	#42
NPY	CCGCTCTGCGACACTACAT	TGTCTCAGGGCTGGATCTCT	#9
Galanin	CAGTTTCTTGCACCTTAAAGAGG	TCTCAGGACTGCTCTAGGTCTTC	#10
Orexin A	CAGACACCATGAACCTTCCTT	GACAGCAGTCGGGCAGAG	#74
CART	AGAAGAAGTACGGCCAAGTCC	CACACAGCTTCCCGATCC	#108
POMC	AGGACCTCACCACGGAAAG	CCGAGAGGTCGAGTCTGC	#62
Mc3R	CCGCCGATAACCATGAACT	GTTAGGCAGCGTCGGATAAG	#18
CRHR1	CCGCTACAACACGACAAACA	TGAGAATCTCCTGGCACTCA	#44
IL-6	CCTGGAGTTTGTGAAGAACAACT	GGAAGTTGGGGTAGGAAGGA	#106
TNF-a	AGAACTCCAGGCGGTGTCT	GAGCCCATTTGGGAACTTCT	#63
NF-kB	ACTGCTCAGGCCCACTTG	TGTCATTATCTCGGAGCTCATCT	#25
SOD1	AGAAACATGGCGGTCCAG	ATGGACACATTGGCCACAC	#5
GRx	TTCCTCATGAGAACCAGATCC	TGAAAGAACCCATCACTGGTTA	#64
GPx	GTTTCCCGTGCAATCAGTTC	GAATTCAGAATCTCTTCATTCTTGC	#2
Catalase	AATGAAGACAACGTCACTCAGG	TGTTCTCACACAGGCGTTTC	#63

NPY, neuropeptide Y; POMC, Pro-opiomelanocortin; CART, Cocaine and amphetamine regulated transcript; Mc3R, Melanocortin receptor 3; IL-6, interlekin-6; TNFα, tumor necrosis factor alpha; NF-κB, nuclear factor kappa-light-chain-enhancer of activated B cells; SOD1, superoxide dismutase 1; GRx, glutathione reductase; GPx; glutathione peroxidase.
