# Supplementary material for: Differential expression of hypothalamic, metabolic and inflammatory genes in response to short-term calorie restriction in juvenile obese- and lean-prone JCR rats
Source: Nutr Diabetes. 2015 Aug 24;5(8):e178–. doi: 10.1038/nutd.2015.28 (PMC4558559; doi:10.1038/nutd.2015.28)
Supplement: Supplementary Table 2 [file nutd201528x2.docx]

Supplementary Table-2: Hypothalamic arcuate nucleus mRNA expression of target genes in the JCR:LA-cp obese-prone and lean-prone rats following caloric restriction (n = 6 rats/group).

|  | Lean-FF | Lean-FR | Obese-FF | Obese-FR |
| --- | --- | --- | --- | --- |
| NPY1R | 1.04 ± 0.13 | 1.18 ± 0.06 | 0.99 ± 0.07 | 0.84 ± 0.12 |
| NPY5R | 1.18 ± 0.19 | 1.16 ± 0.11 | 1.58 ± 0.13 | 1.25 ± 0.24 |
| MCH | 1.05 ± 0.14 | 1.34 ± 0.26 | 1.49 ± 0.21 | 1.14 ± 0.21 |
| Crh | 1.09 ± 0.17 | 1.23 ± 0.16 | 1.37 ± 0.11 | 1.27 ± 0.17 |
| Crhr2 | 1.06 ± 0.15 | 1.19 ± 0.47 | 1.01 ± 0.16 | 0.76 ± 0.16 |
| FAS | 1.02 ± 0.07 | 1.02 ± 0.06 | 1.04 ± 0.07 | 0.87 ± 0.07 |
| ACC | 1.01 ± 0.06 | 1.19 ± 0.06 | 1.2 ± 0.08 | 0.99 ± 0.07 |
| MCD | 1.05 ± 0.15 | 0.96 ± 0.05 | 0.97 ± 0.06 | 1.07 ± 0.04 |
| Cpt1a | 1.01 ± 0.07 | 1.2 ± 0.06 | 1.18 ± 0.06 | 1.05 ± 0.03 |
| Cpt1c | 1.02 ± 0.09 | 1.24 ± 0.11 | 1.21 ± 0.13 | 0.91 ± 0.06 |
| UCP2 | 1.06 ± 0.15 | 1.02 ± 0.08 | 0.99 ± 0.08 | 0.92 ± 0.04 |
| Adipoq | 1.58 ± 0.89^a^ | 11.67 ± 6.27^b^ | 12.25 ± 7.38^b^ | 2.3 ± 1.13^a^ |
| Adipor1 | 1.01 ± 0.05^a^ | 1.19 ± 0.06^b^ | 1.15 ± 0.05^b^ | 0.84 ± 0.02^a^ |
| Adipor2 | 1.04 ± 0.12^a^ | 1.48 ± 0.11^b^ | 1.45 ± 0.13^ab^ | 1.15 ± 0.07^ab^ |
| Il-6r | 1.02 ± 0.08^a^ | 1.12 ± 0.05^a^ | 1.09 ± 0.04^a^ | 0.76 ± 0.04^b^ |
| Th | 1.09 ± 0.18^a^ | 2.55 ± 0.78^a^ | 2.99 ± 1.10^a^ | 3.32 ± 1.14^b^ |
| D1R | 1.19 ± 0.33 | 1.4 ± 0.52 | 1.09 ± 0.42 | 0.65 ± 0.17 |
| D2R | 1.03 ± 0.11 | 1.45 ± 0.38 | 1.33 ± 0.27 | 1.06 ± 0.04 |
| 5HTR | 2.01 ± 0.67^a^ | 2.65 ± 0.46^ab^ | 2.18 ± 0.27^ab^ | 3.97 ± 0.43^b^ |
| TPH2 | 1.1 ± 0.19 | 1.48 ± 0.15 | 1.38 ± 0.30 | 1.34 ± 0.18 |
| CB1R | 1.06 ± 0.16 | 1.22 ± 0.16 | 1.26 ± 0.07 | 1.16 ± 0.08 |
| GR | 1.33 ± 0.46 | 1.15 ± 0.16 | 0.97 ± 0.07 | 0.91 ± 0.05 |
| CLOCK | 1.08 ± 0.13 | 1.19 ± 0.05 | 1.18 ± 0.08 | 1.13 ± 0.06 |
| Arntl | 1.05 ± 0.17 | 1.24 ± 0.13 | 1.15 ± 0.09 | 0.89 ± 0.07 |
| Npas2 | 1.08 ± 0.17 | 1.49 ± 0.18 | 1.45 ± 0.09 | 1.07 ± 0.07 |
| Per1 | 1.04 ± 0.14 | 1.08 ± 0.09 | 0.76 ± 0.04 | 0.93 ± 0.07 |
| Per2 | 1.04 ± 0.12 | 1.09 ± 0.16 | 0.85 ± 0.05 | 1.02 ± 0.15 |
| Cry1 | 1.02 ± 0.09^a^ | 1.0 ± 0.07^a^ | 0.82 ± 0.05^ab^ | 0.67 ± 0.07^b^ |

The values are means ± SEM. One-way ANOVA with post-hoc comparisons using Tukey test was used to detect differences between groups. The row means with different superscripts are significantly different, p < 0.05. NPY1R, neuropeptide Y receptor 1; NPY5R, neuropeptide Y receptor 5; MCH, melanin-concentrating hormone; Crh, corticotropin- releasing hormone; Crhr2, corticotropin- releasing hormone receptor 2; FAS, fatty acid synthase; ACC, Acetyl-CoA carboxylase; MCD, malonyl-CoA decarboxylase; Cpt1a, carnitine palmitoyltransferase 1a; Cpt1c, carnitine palmitoyltransferase 1c; UCP2, uncoupling protein 2; Adipoq, adiponectin; Adipor1, adiponectin receptor 1; Adipor2, adiponectin receptor 2; IL-6R, interleukin-6 receptor; Th, Tyrosine hydroxylase; D1R, dopamine receptor 1; D2R, dopamine receptor 2; TPH2, tryptophan hydroxylase 2; CB1R, cannabinoid receptor 1; GR, glucocorticoid receptor; Clock, Circadian Locomotor Output Cycles Kaput; Arntl, Aryl Hydrocarbon Receptor Nuclear Translocator-Like; Npas2, neuronal PAS domain protein 2; Per 1, period circadian protein homolog 1; Per2, period circadian protein homolog 2; Cry1, Cryptochrome-1; FF, free feeding; FR, food restricted.
